# Frequency of the rs 14035 polymorphism of *RAN* gen in recurrent pregnancy loss: A case-control study

**DOI:** 10.18502/ijrm.v13i5.7156

**Published:** 2020-05-31

**Authors:** Zahrasadat Mortazavifar, Hamidreza Ashrafzadeh, Seyed Morteza Seifati, Nasrin Ghasemi

**Affiliations:** ^1^Medical Biotechnology Research Center, Ashkezar Branch, Islamic Azad University, Ashkezar, Yazd, Iran.; ^2^Abortion Research Center, Yazd Reproductive Sciences Institute, Shahid Sadoughi University of Medical Sciences, Yazd, Iran.

**Keywords:** RAN gene, Repeated abortion, Polymorphism, PCR-RFLP.

## Abstract

**Background:**

Genetic factors could account for recurrent pregnancy loss (RPL). The *RAN* gene is a member of the "large RAS family" and a small GTPase that is essential for the translocation of Ribonucleic acid (RNA) and proteins through the nuclear pore. Mutation in the *RAN* constitutive gene could stop DNA synthesis and alter the expression of genes in the uterus, likely playing a role in recurrent miscarriage.

**Objective:**

The aim was to investigate the frequency of *RAN* (rs 14035) polymorphism in women with RPL compared with women without abortion history.

**Materials and Methods:**

In this case-control study, 100 women with at least two consecutive miscarriages before the 20th wk of gestation and having spouses with karyotype and normal sperm parameters as the case group and 100 women with no history of abortion and having at least one successful pregnancy and normal delivery as the control group. The groups were age matched (20-40 yr). The rs 14035 polymorphism of *RAN* gene was investigated by Polymerase Chain Reaction-Restriction Fragment Length poly morphism technique and the frequency of which was compared between the two groups.

**Results:**

The frequency of TT, TC, and CC genotypes of *RAN* gene polymorphism in the case group were 9%, 40%, and 51%, respectively, and in the control group were 11%, 38%, and 51%, respectively. There was no significant difference in the genotypes between two groups (p = 0.882).

**Conclusion:**

According to our results, it seems that *RAN* polymorphism (rs 14035) is not associated with the risk of RPL in this study population.

## 1. Introduction

Recurrent pregnancy loss (RPL) is one of the most common complications of pregnancy that is defined as two or more consecutive miscarriages occurring one in every 300 pregnancies (1, 2). One possible cause of RPL is impaired miRNA genes processing. The presence of the *RAN* gene polymorphism (rs 14035) may increase the risk of recurrent miscarriages.* RAN* gene is a member of the "large family of RAS" and is a small essential GTPase for the translocation of RNA and proteins through the nuclear pore. It is also involved in mitosis as well as DNA synthesis and promoting the cell cycle. Mutation in the *RAN* gene, which encodes the *RAN*-producing gene, disrupts DNA synthesis and can play a crucial role in abortion (3, 4). Previous studies showed the effect of genetic changes on inducing RPL as a major pregnancy complication. However, there are still so many controversies around these cause and effect (5-9).

miRNAs regulate gene expression at the post-transcriptional stage by suppressing translation or mRNA reduction (10). About 30% of human genes are the targets of protected miRNAs, hence showing that miRNAs are the key regulators in many biological pathways (11). Previous studies have shown a significant relationship between recurrent miscarriage and rs 14035 in women with RPL (12-14).

Since the polymorphism of a gene may be not the same in different populations and geographical areas, this study aimed to determine the frequency and relationship of rs 14035 with RPL in women referred to Yazd Reproductive Sciences Institute.

## 2. Materials and Methods

In this case-control study, blood samples from 100 women aged 20-40 yr with at least two or more idiopathic miscarriages were collected. All cases were referred to the Yazd Reproductive Sciences Institute between March and December 2018.

The control group (n = 100) consisted of 20-40 yr-old women with no history of abortion and having at least one successful pregnancy and normal delivery.

All women with cytogenetic problems, anatomical abnormalities in the reproductive tract, immunological disease, hormonal abnormalities (including thyroid and prolactin disorders), infection, history of cancer, and spouses with abnormal karyotype or impaired semen analysis were excluded.

### DNA extraction 

A total of 200 DNA samples extracted from 2 μl blood specimens from the case and control groups were studied. As previously reported "RNA was isolated from blood samples using the QuantiTectR, RNeasy Micro kit (Qiagen Europe, Germany) according to the manufacturer's instructions. The RNA concentration was measured by NanoDrop spectrophotometer and adjusted to a concentration of 1000 ng/μl. Then, cDNA was synthesized by a RevertAid First Strand cDNA synthesis kit (Thermo Fisher Scientific Inc.), according to the manufacturer's guides. The reverse transcription was performed in 20 μl reactions for 60 min at 42°C, followed by 70°C for 5 min to put the reverse transcriptase out of action. The products of reverse transcription reaction were straightly used in polymerase chain reaction (PCR) in a separate step to amplify the targets (15)".

### Polymerase chain reaction

The final volume of PCR reactions was assumed to be 25 µl in this study so that the mastermix (12.5 µl), DNA (3 µl), forward primers (1 µl), reverse primers (1 µl), and water (7.5 µl) can reach to a volume of 25 µl. It was then mixed in 0.2 microtubes and placed in a thermocycler followed by vortex and microfusion, and based on the temperature program given to the device, the given parts were reproduced. When the PCR reaction was completed, we used electrophoresis to confirm the PCR product and to ensure that it was not contaminated. The microtubes were removed from the apparatus, and 5 µl of the reaction product plated on 2% agarose gel was sampled and subjected to 95 V for 1 hr. The agarose gels were then photographed and analyzed by gel docking. Electrophoresis results were single band in the 152 bp region without any specific band. After completing the samples and examining the products by electrophoresis and ensuring the specific amplification of the PCR product fragment for subsequent steps, it was stored at -20°C. PCR was performed again if the samples lacked bands or had poor bands.

**Figure 1:**
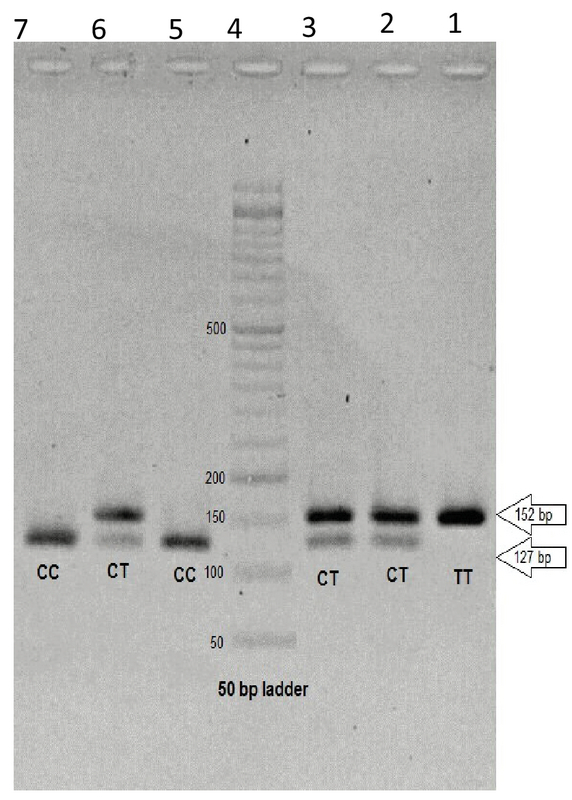
Extracts from the enzymatic digestion of PCR product of polymorphism rs14035. Columns 1, 2, and 3 of the fragments obtained from enzymatic digestion of the healthy sample. Column 4 indicates Ladder. Columns 5, 6, and 7 of the fragments obtained from enzymatic digestion of the patient sample.

After performing PCR reaction for all the patients and controls, the final study was performed by PCR-RFLP (Restriction fragment length polymorphism) technique using BSL1-restricting enzyme. Following the optimization of enzymatic digestion conditions, all samples were subjected to this enzyme and then plated on 2% agarose gel. The optimal temperature for enzymatic digestion of PCR products by the BSL1 enzyme is 55°C. The PCR-RFLP procedure was performed as follows: 18 µl of water was poured into a microtube and then 2 µl of enzyme buffer and 1 µl of enzyme were added. Next, 10 µl of PCR product was added and vertex was performed. The specimens were then incubated in the incubator for 3 hr at 55°C. Finally, 10 µl of the reaction product was applied to 2% agarose gel and exposed to 100 V for 1 hr in order to test the product and then placed in a dock gel to see the result of the gel test (Figure 1).

In the present study, regarding the enzymatic fragmentation status (CCNNNNNNNGG), by conducting PCR-RFLP technique using BSL1 enzyme and performing gel electrophoresis of reaction products, we expected to observe three different patterns as follows:

• In the single-band 152 observation mode after enzymatic digestion, we have the TT allele.
• In the two-bands (127 + 25) observation mode, we have CC allele.
• In the three-bands (252 + 127 + 25) observation mode, we have CT allele.

### Ethical consideration

The research proposal was approved by the Ethics Committee of Yazd Reproductive Sciences Institute, Yazd, Iran (Code: IR.SSU.RSI.REC.1397.024). All participants' information was extracted from their hospital records maintaining confidentiality and entered into the study data collection form.

### Statistical analysis

SPSS software (Statistical Package for the Social Sciences, version 20.0, SPSS Inc, Chicago, Illinois, USA) was used for statistical analysis. Statistical comparisons were performed by Chi-Square and Students' *t* tests. P-value < 0.05 was considered as statistically significant.

## 3. Results

In this study, DNA were extracted from 200 peripheral blood (n = 100/each group). The mean age of the samples in the case and control group were 28.57 ± 4.76, and 27.67 ± 4.09 yr, respectively (p = 0.16). The residential place of case group were 31% from Yazd (n = 31) and 69% (n = 69) was elsewhere (outside the province) (p < 0.001). All of the participants in the control group were from Yazd province (100%).

In the case group, 73 (73%) had a history of abortion in the first trimester of pregnancy, 8 (8%) in the second and third trimesters of pregnancy, and 19 (19%) had a history of abortion in all trimesters. Only 7 (7%) had a history of ectopic pregnancy. Moreover, 9% had a history of stillbirth, 23% a history of partial hydatidiform mole, 25% primary infertility, and 9% had the secondary infertility. In addition, 41% of the couples in the case group had consanguinity and 59% had no consanguineous marriage. There was no significant difference between the genotypic frequency of rs14035 polymorphism in the three CC, CT, and TT genotypes in the case and control groups (p = 0.882) (Table I).

Allele C frequency was 142 (71%) in the women with recurrent miscarriages and 140 (70%) in the control group. The number of allele T in the case group was 58 (29%) and in the control group 60 (30%). Statistical comparison showed no significant difference between the frequency of alleles C and T and the polymorphism studied in the case and control participants (Odds ratio: 0.953; CI: 0.620-1.465; p = 0.826).

The frequency of abortion in the case group (n=100) was as follows: 73 women (73%) had a history of miscarriage in the first trimester, 8 (8%) had a history of miscarriage in second and third trimesters and 19 (19%) had a history of both types of miscarriage. Statistical analysis using the Chi-square test did not show a significant relationship between abortion time and genotype in our participants (p = 0.528) (Table II).

According to the results, no significant relationship was identified between abortion time, history of stillbirth, ectopic pregnancy, and frequency of hydatidiform mole pregnancy with genotypes of the participants (p = 0.528, p = 0.905, p = 0.688, and p = 0.738, respectively) (Table III).

**Table 1:** Distribution of *RAN* (rs14035) polymorphism genotype frequency in the case and control groups


**Genotype**	**Case group (n = 100)**	**Control group (n = 100)**	P-value*
**CC**	51 (51%)	51 (51%)	
**CT**	40 (40%)	38 (38%)	
**TT**	9 (9%)	11 (11%)	<brow>-3</erow> 0.882
*Chi-square test			

**Table 2:** Comparison of the relationship between abortion time and *RAN* (rs14035) polymorphism genotype frequency in the case group (n = 100)


**Genotype/Abortion time**	**First trimester (n = 8)**	**Second and third trimesters (n = 73)**	**First, second, and third trimester (n = 19)**	**P-value**
**TT**	1 (11.1%)	7 (77.8%)	1 (11.1%)	
**CC**	3 (5.9%)	35 (68.6%)	13 (25.5%)	
**CT**	4 (10%)	31 (77.5%)	5 (12.5%)	<brow>-3</erow> 0.528
Chi-Square test, Abortion time				

**Table 3:** Comparison of the relationship between *RAN* (rs14035) polymorphism genotype frequency and history of stillbirth, ectopic pregnancy history, and hydatidiform moles in the case group (n = 100)


	**TT**	**CC**	**CT**	**P-value**
**History of stillbirth (n = 9)**	1 (11.1%)	5 (9.8%)	3 (7.5%)	0.90
**History of ectopic pregnancy (n = 7)**	0 (0%)	4 (7.8%)	3 (7.5%)	0.68
**History of hydatidiform moles (n = 23)**	3 (33.3%)	11 (21.6%)	9 (22.5%)	0.73
Chi-square test				

## 4. Discussion

This study investigated the association of *RAN* polymorphism with the risk of recurrent miscarriage. This gene is involved in biogenesis and miRNA processing and may be involved in the risk of spontaneous abortion. The results of this study showed no association between the frequency of *RAN* (rs14035) gene polymorphism and the risk of recurrent spontaneous miscarriage in the study population.

The biogenesis process of miRNAs is multistep that begins from the cell nucleus and then continues to the cytoplasm through the transcriptional stage. A study by Rah and colleagues have shown that polymorphisms of miRNA-encoding genes may lead to the formation of different secondary structures in miRNAs, which may therefore affect the degree of miRNA stability (16). The miRNAs, small RNA molecules, and encoding affect the pathway of gene expression regulation by interfering with trophoblast cell proliferation, regulating cell proliferation, placental growth, and embryogenesis. In this regard, new insights address the pathologic pathway of idiopathic recurrent miscarriages (17).

To date, few studies have been performed on the role of the *RAN* gene in the human reproductive system. In a case-control study by Jung and colleagues, the association of different types of microRNA biosynthesis system with idiopathic spontaneous abortion was investigated. Using PCR-RFLP technique, they confirmed the association between these polymorphisms and the increased incidence of recurrent miscarriage by examining 238 women in the control group and 338 women with two-time history of idiopathic spontaneous abortion (13). However, their findings were different from those of ours.

In 2017, Fu and colleagues in China explored the relationship between the polymorphisms of genes involved in microRNA biosynthesis (*DICER*, *DROSHA*, and *RAN*) with unexplained recurrent miscarriage in 217 women with a history of miscarriage. These women were compared to 390 healthy unmarried women as the case group by PCR-RFLP technique. Results of this study revealed that although *DICER* rs3742330/*RAN* rs14035 GG/TT + TC combinations were significantly higher in the group with recurrent miscarriage, no significant difference was identified between the two groups (18).

Moreover, Fallah-Sohy and colleagues conducted a case-control study on genotyping of *DICER* gene polymorphism in 20 healthy women and 50 women with RPL using Tetra ARMS (Amplification Refractory Mutation System) PCR technique. They determined *RAN* polymorphism genotype in 10 healthy women and 40 women with unexplained spontaneous recurrent miscarriage by PCR and sequencing method. The results of this study revealed a significant relationship between recurrent spontaneous miscarriage and *RAN* polymorphism (rs14035), but this complication was not correlated with *DICER* polymorphism (rs3742330) (14).

A descriptive study evaluated the association between polymorphism of *RAN* (rs14035) and *XPO5* (rs2257082) genes with recurrent miscarriage in 100 women suffering from recurrent miscarriage and 100 healthy women by PCR-RFLP. The findings demonstrated that *XPO5* gene polymorphism can be a predisposing factor for recurrent miscarriage in pregnant women while the polymorphism of the *RAN* gene appears to have no determinant role (12).

On the other hand, study on miRNA expression in several organs have shown that miRNA expression is specific to each tissue and is abundantly expressed in the placenta (19). "In addition, miRNAs regulate uterine gene expression being associated with inflammatory responses during early implantation and participate in the maternal-fetal immune tolerance (20). Furthermore, there is a report suggesting a link between abnormal miRNA expression and a large number of human reproductive-related diseases (21). One study have also reported genetic polymorphisms being associated with recurrent miscarriage (22). Key molecules involved in miRNA biogenesis such as *XPO5, DROSHA, DICER* have been identified in trophoblast cells thus confirming the biogenesis pathway as being active in the human placenta (23).

In addition, a score of investigations have been performed in Iran and foreign countries on the association of miRNAs with recurrent spontaneous abortion, many of which indicate a significant association between miRNA and recurrent spontaneous abortion.

## 5. Conclusion 

On the basis of our findings, it seems that *RAN* (rs 14035) polymorphism fails to be associated with the risk of spontaneous abortion in the study population.

##  Conflict of Interest

The authors have no conflict of interest to declare.
